# Genome sequences of fifteen soil bacteria isolated from the Dallas-Fort Worth Area, United States

**DOI:** 10.1128/mra.00832-25

**Published:** 2025-11-17

**Authors:** Clayton D. Gabel, Yordanos Kifle, Haley Dahl, Minh Luong, Tuong-Vi Cindy Ngo, Ayesha Asad, Anthony Garcia, Emily Nguyen, Julie Nguyen, Rose Kariampally, Evangeline Theruvath, Andrea Alvarez, Jennifer Bracewell, Aru Chezhian, Naoreen Islam, Selena Nguyen, May Tran, Matthew White, Tahmineh Ebrahimzadeh, Nikesh Kunder, Belle Sharon, Iti Mehta, Kelli L. Palmer

**Affiliations:** 1 Department of Biological Sciences, The University of Texas at Dallas 12335, Richardson, Texas, USA; The University of Arizona, Tucson, Arizona, USA

**Keywords:** genomics, soil microbiology

## Abstract

Soil is a rich source of diverse bacteria that produce useful metabolites, including antibiotics. Here, we report the genomes of 15 bacterial isolates cultured from soils from the Dallas-Ft. Worth metroplex area of the United States, including members of the *Pseudomonas*, *Streptomyces*, *Peribacillus*, *Flavobacterium*, *Enterobacter*, *Rossellomorea*, and *Scandinavium* species.

## ANNOUNCEMENT

Soil is an isolation source for microbes producing metabolites useful for medical and biotechnological applications (1–3). One metabolite class of note is antibiotics, which are staple therapies for infection treatment (4). However, the rise of multidrug resistance in bacteria has limited the current clinical usefulness of many antibiotics (5). The discovery of new antibiotics is paramount for human health.

The Tiny Earth Project crowdsources antibiotic discovery by engaging students in the isolation and characterization of soil bacteria (6). For this study, isolates were collected in the Fall 2021 and 2022 semesters at the University of Texas at Dallas in a course following the Tiny Earth curriculum (7). For each isolate, 1 g of surface soil collected by each student from sites of their choosing in the DFW area was serially diluted with phosphate-buffered saline and plated on agar of the student’s choosing followed by incubation at room temperature for ≤2 weeks. Of the isolates reported here, 13 were obtained from 10% trypticase soy agar soil cultures and two from Reasoner’s 2A agar (R2A). One isolate, UTDF22-5-58, was initially cultured on potato dextrose agar (PDA) but was later regrown on R2A due to low growth yield on PDA. All initial agars contained 25 µg/mL cycloheximide to inhibit fungal growth. Pure cultures of the isolates were obtained by streaking on agar and were cryopreserved in 20% glycerol at −80°C.

To obtain genomic DNA, isolates were revived from cryopreservation by agar and broth culture in previously mentioned media at 37°C overnight. Broth cultures were pelleted by centrifugation and processed with the DNeasy Blood and Tissue Kit (Qiagen) for DNA extraction. DNA integrity was confirmed using 1% agarose gel electrophoresis, and DNA concentrations and purity were assessed using a NanoDrop spectrophotometer (Fisher Scientific).

Illumina HiSeq 2000 sequencing was performed at SeqCenter (Pittsburgh, PA, USA) using the Illumina DNA Prep kit and custom 10 bp unique dual indices with a target insert size of 320 bp. Illumina data were processed using CLC Workbench, v22.0 (Qiagen). Trimming was achieved with a Phred score of 23 and read length maximum of 150 bp. Trimmed reads were then subjected to assembly using the CLC Workbench *de novo* sequencing tool. Contigs < 200 bp were discarded. As assessed by CheckM (v.1.0.18; 8) in Kbase (8), all strains had completeness of Kmers > 95% and contamination <2%, consistent with guidelines outlined previously for high-quality genomes (9–11).

Taxonomic assignments were generated by multiple steps. First, contigs were annotated by RAST (12–14). 16S rRNA genes for each isolate were analyzed for sequence identity with existing sequences via National Center for Biotechnology Information (NCBI) BLASTn (15), giving each isolate a preliminary taxonomic assignment. FastANI (16) in Kbase (8) with comparator genomes in the same species and/or genus was used to further refine taxonomic assignments. Finally, genomes were annotated using the NCBI pipeline PGAP v6.7 (17), and assignments were cross-checked with the automated taxonomy check in NCBI, shown in Table 1.

**TABLE 1:** Genome sequences reported in this study

Strain	Taxonomic assignment	BioSample accession no.	SRA accession no.	Total no. of trimmed reads	Average read depth (x)	Contig N50 (bp)	GenBank shotgun accession no.	No. of contigs	Total length (bp)	GC content (%)
*UTDF21-4AA*	*Pseudomonas soli*	SAMN41405355	SRR33986633	8,290,348	220.49	172,807	JBFLVE000000000	150	5,610,047	63.80%
*UTDF21-AG10*	*Peribacillus frigoritolerans*	SAMN41405356	SRR33986634	7,608,606	205.15	283,086	JBDOCW000000000	96	5,518,351	40.78%
*UTDF21-P1B*	*Scandinavium sp.* ^*a*^	SAMN41405357	SRR33986628	7,304,528	233.97	231,021	JBFLVD000000000	75	4,649,918	55.23%
*UTDF21-P1F*	*Pseudomonas* *putida*	SAMN41405358	SRR33986627	6,214,104	156.88	130,011	JBFLVC000000000	131	5,922,163	61.96%
*UTDF21-EN1*	*Flavobacterium enshiense*	SAMN41405359	SRR33986626	5,530,028	266.08	530,578	JBDOCV000000000	55	3,101,703	38.21%
*UTDF21-JN14*	*Rossellomorea marisflavi*	SAMN41405360	SRR33986639	7,633,352	248.63	301,250	JBDOCU000000000	49	4,574,417	48.68%
*UTDF21-MLA*	*Streptomyces hydrogenans*	SAMN41405361	SRR33986638	7,953,570	136.09	25,669	JBFLVG000000000	1,523	8,726,498	72.39%
*UTDF21-MLD*	*Streptomyces* *virginiae*	SAMN41405362	SRR33986637	7,651,322	137.91	29,777	JBFLVB000000000	1,188	8,233,943	71.65%
*UTDF22-4-2*	*Streptomyces cyaneofuscatus*	SAMN41405363	SRR33986636	5,763,162	101.08	118,477	JBFLVF000000000	298	8,121,031	71.02%
*UTDF22-6-4*	*Enterobacter* *cloacae*	SAMN41405364	SRR33986635	5,022,928	145.02	196,200	JBDOCT000000000	50	5,133,082	55.16%
*UTDF22-16-39*	*Streptomyces cinereoruber*	SAMN41405365	SRR33986633	4,133,666	78.85	76,700	JBDOCS000000000	388	7,615,135	72.10%
*UTDF22-1-47*	*Peribacillus frigoriolerans*	SAMN41405366	SRR33986632	5,103,204	134.66	257,988	JBDOCR000000000	92	5,502,551	41.51%
*UTDF22-12-49*	*Pseudomonas* *putida*	SAMN41405367	SRR33986631	4,963,468	126.17	123,092	JBDOCQ000000000	144	5,805,227	61.55%
*UTDF22-5-58*	*Pseudomonas* *asiatica*	SAMN41405368	SRR33986629	5,422,804	139.41	152,568	JBDOCP000000000	83	5,764,738	62.78%
*UTDF22-5-67*	*Pseudomonas juntendi*	SAMN41405369	SRR33986630	4,883,322	122.58	59,307	JBDOCO000000000	219	5,890,887	61.78%

^*a*^ Initially assigned to *Enterobacter asburiae*. Taxonomic assignment was refined by FastANI and NCBI automated ANI analysis.

It is such that we provide 15 genomes of 13 species of soil bacteria, isolated from the DFW metroplex.

## Data Availability

This Whole Genome Shotgun project and all the SRA raw reads have been deposited to GenBank/NCBI under the Project ID PRJNA1111866.
